# Investigation of Vitamin D-Binding Protein Polymorphism Impact on Coronary Artery Disease and Relationship with Longevity: Own Data and a Review

**DOI:** 10.1155/2016/8347379

**Published:** 2016-04-06

**Authors:** Donatas Stakisaitis, Vita Lesauskaitė, Milda Girdauskaitė, Ernestas Janulionis, Albertas Ulys, Rimantas Benetis

**Affiliations:** ^1^Laboratory of Cancerogenesis and Cancer Epidemiology, Scientific Research Center, National Cancer Institute, Santariskiu 1, LT-08660 Vilnius, Lithuania; ^2^Department of Biolaw, Mykolas Romeris University, Ateities 21, LT-08303 Vilnius, Lithuania; ^3^Department of Geriatrics, Medical Academy, Lithuanian University of Health Sciences, A. Mickevičiaus 9, LT-44307 Kaunas, Lithuania; ^4^Radiation and Medical Oncology Clinics, National Cancer Institute, Santariskiu 1, LT-08660 Vilnius, Lithuania; ^5^Oncosurgery Clinics, National Cancer Institute, Santariskiu 1, LT-08660 Vilnius, Lithuania; ^6^Institute of Cardiology of the Medical Academy, Lithuanian University of Health Sciences, Sukileliu 17, LT-50161 Kaunas, Lithuania

## Abstract

The aim of the study was to assess the effect of vitamin D-binding protein (DBP) polymorphism on coronary artery disease (CAD). DBP phenotypes were identified in the groups: control (*n* = 306), men suffering from CAD (*n* = 154), and long-lived individuals (*n* = 108). Isoelectric focusing of DBP phenotypes in serum was performed on polyacrylamide gel. Distribution of DBP phenotypes in the study groups was found to be in Hardy-Weinberg equilibrium. Gc1s-1s phenotype and Gc1s allele frequency in CAD groups were significantly higher than in control, and Gc1s allele frequency was found significantly more often in CAD compared with long-lived group (*p* < 0.05). The Gc2 allele frequency in control was higher as compared with Gc2 frequency in CAD group (*p* < 0.05). The Gc2-2 phenotype was more frequent in long-lived survivors than in the CAD group (*p* < 0.05). It was found that the Gc1s allele significantly increased the risk of CAD with the odds ratio (OR) equal to 1.45 (*p* < 0.02) and showed Gc2 to be related with a decreased risk of CAD (OR = 0.69; *p* < 0.03). Authors review the role of DBP in resistance to atherosclerosis and cancer as the main longevity determinants.

## 1. Introduction

Long-lived individuals are an important antiatherogenic control group for the evaluation of genetic markers in the pathogenesis of atherosclerosis. The longevity genetic markers in the Lithuanian population were found to be the low level of apolipoproteine B (apoB) and the apolipoproteine E (apoE), the low apoB/apoA-1 ratio, and the *d* gene responsible for dry cerumen [1, 2]. The* PI* ^*∗*^ *Z* gene of the alpha-1-proteinase inhibitor was significantly more frequent in patients with coronary artery disease (CAD) than in long-lived individuals [3], and the 0 blood group can serve as a protective antiatherogenic factor in women; in long-lived individuals, the B blood group was significantly more rare than in healthy population and in CAD patients in the Lithuanian population [4].

The aging process and mortality are associated with the insufficiency of vitamin D: the low serum 25-hydroxyvitamin D (25(OH)D) level has been linked to all-cause, cardiovascular, cancer and infection-related mortality [5]. An optimal concentration of vitamin D delaying the aging phenomenon and low 25(OH)D concentrations might be a marker for a poor health status related to premature mortality [6]. The lower 25(OH)D levels observed in females as compared with males play a more relevant role in conditioning the severity of CAD; in males, vitamin D status was independently related to the prevalence of CAD [7]. The joint effect of low serum 25(OH)D and low DBP levels is associated with the risk of frailty, and serum DBP levels affect the 25(OH)D-frailty relationship in older men [8].

The major transporter of vitamin D metabolites in blood circulation is the multifunctional plasma vitamin D-binding protein (DBP), also known as a human group specific component (Gc) [9]. The plasma 25(OH)D and DBP levels are related to the DBP phenotype [10, 11]. The DBP has a significantly lower affinity constant for 25(OH)D as compared with the Gc1s or Gc1f isoforms [9].

The* Gc *gene, which encodes the DBP, maps to chromosome 4q12-q13 [12]. There are three common phenotypic alleles in the DBP, Gc1s (slow), Gc1f (fast), and Gc2, differing by combinations of two nonsynonymous single-nucleotide polymorphisms (SNPs), rs4588 and rs7041, which differ by amino acid substitutions and by their glycosylation pattern in the DBP (galactose and sialic acid in both Gc1s and Gc1f; galactose only in Gc2); rs7041 (G→T) encodes for the glutamic acid to aspartic acid change, while rs4588 (C→A) encodes for the threonine to lysine change. The DBP variants Gc1s, Gc1f, and Gc2 are a haplotypic combination of rs7041 and rs4588, where Gc1s = rs7041 (G) and rs4588 (C), Gc1f = rs7041 (T) and rs4588 (C), and Gc2 = rs7041 (T) and rs4588 (A). The combination of the 3 DBP variants results in six common phenotypes [13–15] (Table 1).

Plasma samples from patients and healthy volunteers confirmed a significantly higher concentration of DBP in atherosclerotic subjects [16]. No differences in DBP phenotype frequencies of the* Gc* gene polymorphisms and in serum DBP levels between type 1 diabetic patients and control subjects were found, whereas the association between* Gc *gene polymorphism and patients with type 2 diabetes mellitus has produced conflicting results [17, 18].

The DBP serum level may be a biomarker for a vascular injury having important prognostic and diagnostic implications [19]. The DBP also plays an important role in the response to tissue injury as DBP can be converted to a macrophage-activating factor [20]. Another role of DBP in response to injury is to scavenge for vascular and extracellular actin as a result of cellular necrosis, and DBP has been shown to be present in lower circulating concentrations in inflammatory or necrotic processes [21–23]. DBP serves as a growth factor for vascular smooth muscle cells [24], and it has been found in thrombotic plaques of coronary arteries [25].

Comparing donors, patients with CAD, and long-lived individuals, the Gc1s allele was significantly most frequent in CAD study groups. Comparing CAD patients and long-lived individuals, the Gc2-2 phenotype was significantly more frequent in the group of long-lived individuals. Authors discuss the possible mechanisms of atherogenesis and other important longevity factors in relationship with DBP polymorphism.

## 2. Materials and Methods

The DBP phenotypes have been identified in the following groups of Lithuanian population individuals: control (blood donors; *n* = 306; aged 19 to 52 years); common men suffering from CAD (aged 28 to 72 years) (coronary atherosclerosis plaques were diagnosed by coronarography and during coronary bypass surgery; CAD group was formed with the exclusion criteria for type 1 and type 2 diabetes mellitus); long-lived individuals with no data of myocardial infarction (*n* = 108; men aged 85 to 102 years (*n* = 38), women aged 90 to 103 years (*n* = 70)). The study groups contained only Caucasian (white) population. The institutional and ethical approval of the study was obtained. The isoelectric focusing of DBP phenotypes was performed on thick polyacrylamide gel with ampholytes pH 3.5–5.0, 4.5–6.5, and 4.2–5.0. We assessed the combined effects of two known polymorphisms in the* Gc* gene (rs4588 and rs7041) composing the alleles Gc1s, Gc1f, and Gc2 on CAD risk and relationship with longevity. Statistical analysis was performed using SPSS 21.0 and the R project 2.8.1. Frequency of DBP phenotypes was calculated with reference to Hardy-Weinberg equilibrium. Categorical data are expressed as frequencies and percentages. The normal approximation method was used to calculate 95% confidence intervals for DBP allele frequencies. Chi-square test was used to test for the Hardy-Weinberg equilibrium and comparison of DBP phenotype frequencies among the groups. We calculated the relative risk (measured as an odds ratio (OR)) and the corresponding 95% confidence intervals (95% CI) to find out the association of DBP alleles with CAD and longevity. The result was considered significant at the *p* value < 0.05.

In the literature review we included DBP relationship with atherogenesis and cancer while resistance to cancerogenesis is an additional important determinant for longevity. The literature retrieval was accessed through PubMed (1986–2015) using the terms of vitamin D-binding protein, atherosclerosis, and longevity, including the appropriate Boolean operators “AND” and “OR.”

## 3. Results

### 3.1. DBP Phenotype Frequency in the Study Groups

The frequency of DBP phenotypes in the population (control group) was as follows: Gc1s-1s, 38.2%; Gc1s-1f, 9.8%; Gc1s-2, 38.9%; Gc1f-1f, 0.3%; Gc1f-2, 5.5%; Gc2-2, 7.2% (Table 2). The distribution of the DBP phenotypes in all study groups was found to be in the Hardy-Weinberg equilibrium.

The CAD group Gc1s-1s phenotype frequency was significantly higher compared with the Gc1s-1s phenotype frequency in the control group (*p* < 0.02).

As to the frequency of the Gc2-2 phenotype between the CAD and long-lived groups, the Gc2-2 phenotype was significantly more frequent in the long-lived group (*p* < 0.02).

A significant difference of Gc2-2 phenotype frequency was found between the control and long-lived groups (*p* < 0.04).

### 3.2. DBP Polymorphism in CAD and Long-Lived Survivors Groups

In the control and in patients with CAD and long-lived individuals the Gc1s allele was most frequent: it was statistically more frequent than the Gc1f and Gc2 alleles (*p* < 0.05; Table 3).

The Gc1s allele frequency in the CAD group was higher than in the control group (*p* < 0.02). The difference of the Gc1s allele frequency in the long-lived group compared with the CAD group was also significant, being more frequent in CAD patients (*p* < 0.05).

The Gc2 allele in the control was significantly more frequent than in the CAD group (*p* < 0.03). The Gc2 allele frequency in the CAD group compared with the long-lived group was statistically significant; the Gc2 allele was more frequent in the long-lived group (*p* < 0.02).

The Gc1s allele was found to significantly increase the risk of CAD (OR 1.45; CI 95% = 1.08–1.95, *p* < 0.02). Also, the OR showed that the Gc2 allele was related to a decreased risk of CAD (OR = 0.69; CI 95% = 0.50–0.95, *p* < 0.03). No statistical significance of Gc1f allele carriers in association with CAD was found (OR = 0.84, CI 95% = 0.49–1.43, *p* > 0.05). Based on the results of OR, the Gc1s allele neither increases nor decreases longevity (OR = 1, CI 95% = 0.72–1.37, *p* > 0.05). Statistically not significant results were obtained with the Gc1f carriers in long-lived survivors (OR = 0.62, CI 95% = 0.31–1.21, *p* > 0.05).

The study of the association between CAD and DBP phenotypes has shown that the Gc1s-1s phenotype statistically increases the risk of coronary atherosclerosis (OR = 1.66, CI 95% = 1.12–2.45, *p* < 0.02). A significant relationship between the Gc2-2 phenotype and longevity was revealed (OR = 2.08, CI 95% = 1.04–4.18, *p* < 0.04). No significant statistical association of OR among the other DBP phenotypes was found both in the CAD group and in the long-lived survivors group.

## 4. Discussion

Protection from atherosclerosis and resistance to cancer are the main longevity reasons. Long-lived survivors as the control cohort are important for evaluating the role of genetic determinants in atherogenesis, cancerogenesis, and other diseases related to mortality. Recent studies show that an optimal concentration of vitamin D is important in delaying aging-related phenomena and acts as a protective factor against atherogenesis and cancer-related mortality. According to the meta-analysis of prospective cohort studies from Europe and the United States, the lowest quintile of serum 25(OH)D concentration was associated with increased all-cause and cardiovascular mortality and an association with cancer mortality observed in subjects with a history of cancer [6]. Several atherosclerosis-related common aging-associated diseases such as osteoporosis, hypertension, and diabetes are known to be vitamin D-dependent [26].

The atherosclerosis development is a multistep process associated with aging. Atherosclerosis and chronic inflammatory mechanisms involved in a chronic disease are in CAD coexistence [27]. Overweight and obesity resulting in clinical conditions such as the metabolic syndrome, early atherosclerosis, dyslipidemia, hypertension, and type 2 diabetes mellitus, all leading to high mortality rates in young adults, change the current increasing trend of worldwide longevity [28]. The risk factors associated with the leading causes of death from cancer (lung, renal, colorectal, breast, uterus, stomach, and liver) include also overweight/obesity [29, 30]. The further change in mortality patterns will accompany success in the reduction of the number of mortalities attributable to degenerative conditions such as CAD and cancer [31].

### 4.1. DBP and Long-Lived Survivors

The study data suggest that the multifunctional plasma protein DBP, a major transporter of vitamin D metabolites in the circulation, could be one of the genetic determinants related to longevity. There are three common codominant phenotype alleles known as Gc1s, Gc1f, and Gc2, differing by amino acid substitutions as well as glycosylation [13]. Gc2 is glycosylated with a terminal galactose, whereas Gc1s and Gc1f contain both galactose and sialic acid [32]. From widespread use in population genetics, the frequency of Gc2 is known to be highest among whites and lowest among black Africans, whereas the opposite is true for Gc1f [33]. The plasma concentration of DBP depends on the DBP phenotype being highest in Gc1-1 (Gc1s-1s, Gc1s-1f, and Gc1f-1f), intermediate in Gc1-2 (Gc1s-2, Gc1f-2), and lowest in Gc2-2 [34, 35]. The genetic variation of DBP has the potential to alter serum 25(OH)D concentrations.* In vitro* data have shown that the Gc2 allele has a significantly lower affinity constant for 25(OH)D compared to the Gc1s or Gc1f alleles [9, 36]. Individuals with two copies of the Gc2 allele (Gc2-2) have significantly lower 25(OH)D serum concentrations compared with other DBP phenotypes [10, 11, 37]. DBP concentration and DBP phenotypes were significant predictors of 25(OH)D concentration, even after adjustment for the effects of the season, sunbathing habits, skin thickness, the use of vitamin supplements, smoking, and body mass index [35].

In the study, CAD group was formed of patients with exclusion criteria of type 1 and type 2 diabetes mellitus. The meta-analysis demonstrated that the DBP polymorphism was moderately associated with an increased susceptibility to type 2 diabetes mellitus in Asians, but no such association was found in European populations [17]. No differences in DBP phenotype or allele frequencies of the DBP polymorphism and in serum DBP levels between type 1 diabetic patients and control subjects were found [18].

The study data revealed the Gc2 allele and the Gc2-2 phenotype to be statistically more frequent in the long-lived group as compared with the CAD group in the Lithuanian population. A significant relationship between the Gc2-2 phenotype and longevity was revealed (OR = 2.08). In the literature, we have found no more data related to DBP polymorphism studies in the long-lived survivors.

### 4.2. Atherogenesis, Vitamin D, and Genetic Markers of Longevity

The study data show a significant increase of the Gc1s allele and Gc1s-1s phenotype frequency in the CAD patient groups compared with the control and long-lived individuals in the Lithuanian population. The Gc2 allele could be a marker of resistance to atherogenesis: the Gc2 allele in the control and in long-lived groups was significantly more frequent than in the CAD groups.

The DBP plasma level may be a biomarker for vascular injury, which will have important prognostic and diagnostic implications [19]. Besides serving as a transporter of vitamin D, DBP also plays an important role in response to tissue injury, in which DBP can be converted to a macrophage-activating factor (DBP-MAF) stimulating macrophages [20]. Another role of DBP in response to injury is to scavenge for vascular and extracellular actin as a result of cellular necrosis, and DBP has been shown to be in lower circulating concentrations in the presence of inflammatory or necrotic diseases [21–23]. DBP both has a chemotactic function and serves as a growth factor for vascular smooth muscle cells [24]. DBP has been found in thrombotic plaques of coronary arteries [25]. Patients with acute coronary syndromes have a persistent elevation of plasma DBP over 6 months as compared to healthy volunteers [38]. Plasma samples from patients and healthy volunteers confirmed a significantly higher concentration of DBP in atherosclerotic subjects [16]. On the contrary, other investigators found decreased levels of DBP in the plasma of patients statistically correlated with the number of affected coronary arteries [39]. Human studies evaluating the relationship between 25(OH)D and apoA-I and HDL-cholesterol (HDL) mostly suggest a positive link between increasing 25(OH)D levels and plasma apoA-I and HDL, whereas high 25(OH)D levels provide enhanced atheroprotection [40]. High plasma levels of HDL have protective effects on atherosclerosis and CAD [41, 42]. HDL biomarkers (HDL, ApoA-I) and low-density-lipoprotein (LDL) cholesterol biomarkers (LDL, Apo B) were directly associated with 25(OH)D; HDL-cholesterol biomarker levels were positively associated with higher 25(OH)D levels, and such relationship was stronger than those of LDL or free cholesterol [42, 43]. 25(OH)D deficiency was associated with increased apoB among children [44]. In middle-aged and older men, the low serum 25(OH)D concentration was associated with an increased risk of death mainly in those with a lower magnesium intake [45].

Investigations in the Lithuanian population revealed that the low serum apoB level and especially low apoB/apoA-I may be longevity markers. Another longevity marker was found to be dry cerumen. A significant increase in the frequency of the *d* gene responsible for dry cerumen in long-lived individuals was found as compared with the control. The ratio apoB/apoA-1 was higher in the donors with a humid ear wax (*w* gene) than in those with the dry variant [1]. The* PI* ^*∗*^ *Z* gene of the alpha-1-proteinase inhibitor was significantly more frequent in patients with coronary atherosclerosis than in long-lived survivors, and the local as well as systemic inactivation of the alpha-1-proteinase inhibitor in the atherosclerotic process could be related to hyperlipidemia: congenital (alpha-1-proteinase inhibitor deficiency, hyperlipidemia) and acquired (related to smoking, chronic inflammatory diseases, aging) imbalance of the proteinase-antiproteinase system is considered to be one of the atherogenic factors [3]. The B blood group can be related to coronary atherosclerosis, while the 0 blood group can possibly serve as a protective antiatherogenic factor in women in the Lithuanian population. In the long-lived individuals, the frequency of the B group was significantly rarer than in the healthy control [4]. The Lithuanian population showed a very strong negative correlation of the apoE serum level in the control group consisting of young to long-lived individuals [2]. It has been demonstrated that apoE has a major impact on longevity, and apoE may also play a role in other pathological conditions including cancer, rheumatoid arthritis, and macular degeneration [46, 47].

### 4.3. Carcinogenesis and DBP

Aging and cancer are tightly associated phenomena. Accumulation of damage on DNA and telomeres causes both aging and cancer; moreover, the signaling pathways seem to converge on tumor suppressor protein, p53, which seems to be regulated by vitamin D. Also, telomerase reverse transcriptase might be molecular mechanisms mediating the vitamin D action in aging and cancer [26]. In elderly women, lower serum 25(OH)D concentrations appear to be an independent risk factor for cancer-specific mortality [48]. A decrease in the serum vitamin D level is one of the risk factors for the development and progression of renal cell carcinoma, and 25(OH)D may prevent carcinoma [49]. Men with higher blood serum DBP concentrations were at a significantly decreased risk of kidney cancer [50]. Men with higher 25(OH)D concentrations and serum DBP below the median (the DBP heterozygote) were associated with an elevated risk of pancreatic cancer [51]. Genotyping for rs7041 and rs4588 polymorphism showed that subjects with the DBP phenotype Gc1f-1f had 23–26% reduced risk of cancer incident as compared with the Gc1s-1s and Gc2-2 phenotypes [52]. The Gc2 allele and Gc2-2 phenotype of the DBP are associated with a decreased postmenopausal breast cancer risk independent of the vitamin D status. It was hypothesized that women carrying the Gc2 allele(s) have a higher uptake of the DBP–25(OH)D complex or a better transport to the target organs, and different DBP glycosylation patterns in the Gc2 allele may explain the observed reduced breast cancer risk [37]. Inverse associations have also been shown between the serum 25(OH)D level and breast cancer development, risk for breast cancer recurrence, and mortality in women with early-stage breast cancer [53]. A prospective cohort study shows that the prostate cancer risk has been increased when circulating 25(OH)D and DBP concentrations are elevated [54]. It was noted that higher serum DBP concentrations may sequester more 25(OH)D and reduce free 25(OH)D bioavailability. A simultaneous examination of DBP phenotypes DBP and 25(OH)D levels may be important in determining the association of vitamin D with cancer risk [51]. Furthermore, female life expectancies exceed those of males by six or more years [31].

### 4.4. Osteoporosis, DBP, and Aging

Osteoporosis is a common feature of the aging process, coexisting with atherosclerosis and its risk group individuals. Many other factors, including the treatment of cancer, result in secondary osteoporosis [55]. The vast majority of efforts thus far have focused on bone loss in patients with breast and prostate cancer [56]. Furthermore, marked improvements in survival for many cancers mean that strategies to limit bone loss and reduce fracture risk must be incorporated into the care plans for nearly all cancer patients. The high frequency of prostate cancer-related metastatic bone disease has prompted considering this pathway as a therapeutic target [57].

As a candidate gene for osteoporosis, the DBP was studied. Investigators found a highly significant difference in the premenopausal bone fracture risk among women with Gc2-2 and a low risk of bone fractures, three times lower with Gc2-2 as compared with Gc1-1 [58]. The serum DBP is the precursor for the DBP-MAF [59]. It was hypothesized that differences between the DBP phenotypes concerning DBP-MAF might be either qualitative or quantitative: the differences in amino acid sequence among DBP types could theoretically result in a qualitative difference between Gc1-MAF and Gc2-MAF. Gc1 is glycosylated with both galactose and sialic acid, whereas Gc2 carries only the galactose residue [32]; this is of interest, because the conversion of DBP into the DBP-MAF involves removal of galactose and sialic acid residues from DBP by the actions of *β*-galactosidase and sialidase enzymes associated with the membranes of B- and T-lymphocytes, respectively [60]. Additionally, the higher mean plasma level of DBP with Gc1-1 compared with Gc2-2 results in a larger quantity of substrate for DBP-MAF synthesis in subjects with Gc1-1 and in that way perhaps in a higher basal level of DBP-MAF. The activated DBP in the form of DBP-MAF plays a role in bone modeling/remodeling and thereby has an effect on the risk of bone fractures. The DBP phenotype may be an important predictor of premenopausal bone fracture in whites and suggests that DBP plays an active pathophysiological role in the activity of osteoclasts [58]. Others have demonstrated that the genetic effect of the* Gc* gene on fracture risk appears only in combination with other genetic and environmental risk factors for bone metabolism [61].

## 5. Conclusion

The role of DBP polymorphism in atherogenic and cancerogenic processes, in combination with other genetic determinants and risk factors, as well as in the cohorts free of the other genetic and risk factors, and vitamin D effects in longevity mechanisms is an important aging research area.

## Figures and Tables

**Table 1:** *Gc* gene SNPs and DBP genetic polymorphism.

DBP system		
SNPs of *Gc *gene	Alleles	Genotypes/phenotypes
rs7041 T (Asp) rs4588 C (Thr)	Gc1f	Gc1f-1f Gc1f-1s Gc1f-2 Gc1s-1s Gc1s-2 Gc2-2
rs7041 G (Glu) rs4588 C (Thr)	Gc1s	
rs7041 G (Glu) rs4588 A (Lys)	Gc2	

**Table 2:** DBP phenotype frequency in the studied groups.

Groups	*n*	DBP phenotype					
		Gc1s-1s *n* (%)	Gc1s-1f *n* (%)	Gc1s-2 *n* (%)	Gc1f-1f *n* (%)	Gc1f-2 *n* (%)	Gc2-2 *n* (%)
Control	306	117 (38.2)	30 (9.8)	119 (38.9)	1 (0.3)	17 (5.5)	22^*∗∗*^ (7.2)
CAD	154	78^†^ (50.6)	14 (9.1)	48 (31.2)	1 (0.7)	5 (3.2)	8 (5.2)
Long-lived	108	45 (41.7)	8 (7.4)	37 (34.3)	0	3 (2.8)	15 (13.9)^*∗*^

^*∗*^Gc2-2 phenotype frequency in the long-lived compared with the CAD group (*p* < 0.02).

^*∗∗*^Gc2-2 phenotype frequency in the control compared with the long-lived group (*p* < 0.04).

^†^Gc1s-1s phenotype frequency in the CAD compared with the control group (*p* < 0.02).

**Table 3:** DBP alleles frequency in the studied groups.

Groups	Total alleles *n*	DBP alleles frequency (%; 95% CI)			*p* ^*∗*^
		Gc1s	Gc1f	Gc2	
Control	612	62.6 (58.8–66.4)	8.0 (5.9–10.1)	29.4 (25.8–33.0)	<0.05
CAD	308	70.8 (65.7–75.8)^*∗∗*^	6.8 (4.0–9.6)	22.4 (17.7–27.0)^†^	
Long-lived	216	62.5 (56.0–69.0)^*∗∗∗*^	5.1 (2.2–8.0)	32.4 (26.2–38.6)^‡^	

^*∗*^Gc1s allele frequency compared with Gc1f or Gc2 alleles frequency in the study groups.

^*∗∗*^Gc1s allele frequency in the CAD compared with the control group (*p* < 0.02).

^*∗∗∗*^Gc1s allele frequency in the long-lived compared with the CAD group (*p* < 0.05).

^‡^Gc2 allele frequency in the long-lived compared with CAD group (*p* < 0.02).

^†^Gc2 allele frequency in the CAD compared with the control group (*p* < 0.03).
